# Pathways that Regulate ROS Scavenging Enzymes, and Their Role in Defense Against Tissue Destruction in Periodontitis

**DOI:** 10.3389/fphys.2017.00351

**Published:** 2017-05-30

**Authors:** Hiroyuki Kanzaki, Satoshi Wada, Tsuyoshi Narimiya, Yuuki Yamaguchi, Yuta Katsumata, Kanako Itohiya, Sari Fukaya, Yutaka Miyamoto, Yoshiki Nakamura

**Affiliations:** ^1^Maxillo-Oral Disorders, Tohoku University HospitalSendai, Japan; ^2^Department of Orthodontics, School of Dental Medicine, Tsurumi UniversityYokohama, Japan

**Keywords:** oxidative stress, cytoprotective enzymes, ROS, osteoclast, periodontitis

## Abstract

Periodontitis, an inflammatory disease that affects the tissues surrounding the teeth, is a common disease worldwide. It is caused by a dysregulation of the host inflammatory response to bacterial infection, which leads to soft and hard tissue destruction. In particular, it is the excessive inflammation in response to bacterial plaque that leads to the release of reactive oxygen species (ROS) from neutrophils, which, then play a critical role in the destruction of periodontal tissue. Generally, ROS produced from immune cells exhibit an anti-bacterial effect and play a role in host defense and immune regulation. Excessive ROS, however, can exert cytotoxic effects, cause oxidative damage to proteins, and DNA, can interfere with cell growth and cell cycle progression, and induce apoptosis of gingival fibroblasts. Collectively, these effects enable ROS to directly induce periodontal tissue damage. Some ROS also act as intracellular signaling molecules during osteoclastogenesis, and can thus also play an indirect role in bone destruction. Cells have several protective mechanisms to manage such oxidative stress, most of which involve production of cytoprotective enzymes that scavenge ROS. These enzymes are transcriptionally regulated via NRF2, Sirtuin, and FOXO. Some reports indicate an association between periodontitis and these cytoprotective enzymes' regulatory axes, with superoxide dismutase (SOD) the most extensively investigated. In this review article, we discuss the role of oxidative stress in the tissue destruction manifest in periodontitis, and the mechanisms that protect against this oxidative stress.

## Introduction

Periodontitis is a common disease worldwide of the tissues surrounding the teeth, and is caused by bacterial infection. It is characterized by a dysregulation of the host inflammatory response, which eventually results in soft and hard tissue destruction (Mercado et al., 2003; Bartold et al., 2005). Tissue destruction in periodontitis is considered to result from an excessive inflammatory response to bacterial plaque. This leads to release of reactive oxygen species (ROS), such as hydrogen peroxide and superoxide, from neutrophils (Waddington et al., 2000; Canakci et al., 2005; Dahiya et al., 2013; Miricescu et al., 2014; Callaway and Jiang, 2015; White et al., 2016), which results in oxidative stress—an imbalance between the production of ROS and antioxidant defenses.

Generally, ROS produced from immune cells exhibit an anti-bacterial effect and play a role in host defense and immune regulation (Baehner et al., 1975; Canakci et al., 2005). Excessive ROS however, can exert cytotoxic effects (Esterbauer et al., 1991), cause oxidative damage to proteins and DNA (Wells et al., 2009), can interfere with cell growth and cell cycle progression (Chang et al., 2013), and induce apoptosis (Yu et al., 2012) of gingival fibroblasts. This collectively enables ROS to directly induce periodontal tissue damage. Some ROS are also indirectly involved in periodontal tissue destruction through their role as intracellular signaling molecules in the osteoclastogenic pathway (Ha et al., 2004), and indeed, excessive activation of osteoclasts is a typical pathology in severe periodontitis.

Cells normally have several protective mechanisms against these oxidative stressors (Furukawa-Hibi et al., 2005; Kensler et al., 2007; Hsu et al., 2008), most of which involve the induction of cytoprotective enzymes that scavenge ROS (Mates and Sanchez-Jimenez, 1999). One protective mechanism involves nuclear factor E2-related factor 2 (NRF2), a master regulatory transcription factor for the synthesis of cytoprotective enzymes. NRF2 has been reported to be a negative regulator of osteoclastogenesis (Kanzaki et al., 2013, 2014, 2015).

**Figure 1:**
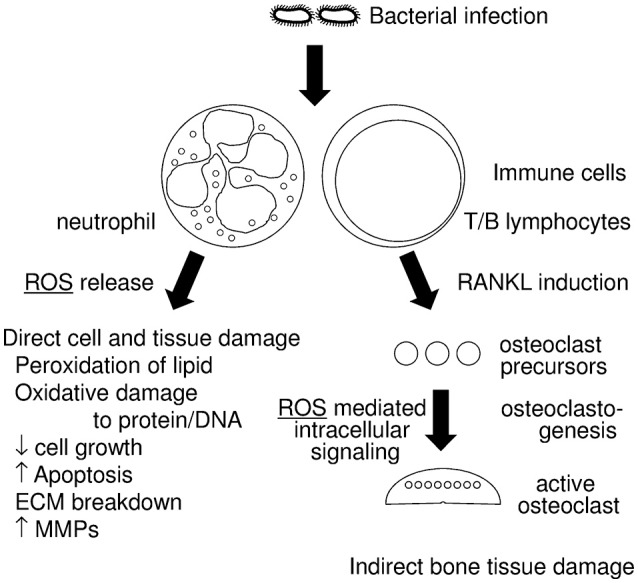
Diagram of the relationship between periodontitis and reactive oxygen species (ROS). Infections with periodontopathogenic bacteria induce host immune responses, which includes neutrophil and T/B lymphocyte activation. Activated neutrophils generate ROS that have an anti-bacterial effect and are thus a form of host defense. Excessive ROS, however, result in direct cell and tissue damage, which includes (1) peroxidation of lipids, (2) oxidative damage to protein and DNA, (3) decrease of cell growth, (4) increased apoptosis, (5) breakdown of the extracellular matrix (ECM), (6) induction of matrix metalloproteases (MMPs). Activated T/B lymphocytes induce RANKL in periodontal tissue, which results in the augmentation of osteoclastogenesis. Some ROS work as intracellular signaling molecules in RANKL stimulation.

The relationship between periodontitis and ROS is illustrated in Figure 1. In this review article, we will discuss; (1) the role of oxidative stress in tissue destruction in periodontitis, and (2) mechanisms that protect against oxidative stress.

## The role of oxidative stress in the tissue destruction in periodontitis

Increased ROS are a hallmark of inflammation induced by neutrophils against invading bacteria, and are involved in tissue destruction (Sheikhi et al., 2000, 2001; Chapple et al., 2007; Matthews et al., 2007a). Periodontal tissue destruction is caused, in part, by neutrophils exhibiting an excessive inflammatory response to bacterial plaque (Canakci et al., 2005; Miricescu et al., 2014), with a high volume of ROS also generated by neutrophils in periodontal tissues with chronic periodontitis (Matthews et al., 2007b). ROS production in neutrophils is driven, in part, by nicotinamide adenine dinucleotide phosphate oxidase (NOX) and the purine degradation pathway, which is significantly accelerated in inflamed periodontal tissue (Giannopoulou et al., 2008; Barnes et al., 2009).

The release of ROS plays a critical role in periodontitis-associated tissue destruction, where ROS exert both direct and indirect effects on bone destruction. Directly, it can induce cytotoxic effects and oxidative damage to proteins and DNA. It can also interfere with cell growth and cell cycle progression (Chang et al., 2013), induce apoptosis (Yu et al., 2012) of gingival fibroblasts, and cause matrix degradation via the induction of matrix proteinases. Indirectly, ROS acts as an intracellular signaling molecule during osteoclastogenesis; an important process in hard tissue degeneration.

ROS exert cytotoxicity, such as peroxidation of lipids and phospholipids, against cells as well as the extracellular matrix (ECM). Additionally, protein aggregation through ROS-mediated oxidation (Squier and Bigelow, 2000; Squier, 2001; Friguet, 2002) leads to the breakdown of cell/tissue homeostasis (Hohn et al., 2014). ROS can also stimulate ECM degradation by inducing the breakdown of glycosaminoglycan (Fuchs and Schiller, 2014) and matrix proteinases (Dasgupta et al., 2009, 2010; Kar et al., 2010).

In addition to having a direct cytotoxic effect, ROS can indirectly promote hard tissue degeneration through their role in osteoclastogenesis. Intracellular signaling molecules that mediate osteoclastogenesis, a signaling cascade central to the destruction of alveolar bone, include ROS (Bax et al., 1992; Ha et al., 2004). In hard tissue destruction, alveolar bone resorption is driven by osteoclasts, the tissue-specific macrophage polykaryon created by the differentiation of monocyte/macrophage precursors cells on the bone surface (Teitelbaum, 2000). Generation of osteoclasts requires physical contact between osteoclast precursor cells and specific mesenchymal cells, such as marrow stromal cells or osteoblasts (Udagawa et al., 1990). The key osteoclastogenic cytokine receptor activator of nuclear factor-kB ligand (RANKL) is a membrane-bound protein on osteoblasts and their precursors, which is recognized by the receptor, RANK, on marrow macrophages, thus prompting them to differentiate into osteoclasts (Lacey et al., 1998; Yasuda et al., 1998). In normal physiological circumstances, RANKL is principally expressed by mesenchymal cells of the osteoblast lineage, but in states of skeletal inflammation (Kong et al., 1999), as well as in periodontitis (Kawai et al., 2006), RANKL is produced in abundance by lymphocytes. Other key factors involved in osteoclastogenesis include TRAF6, RAC1, and NOX (Abo et al., 1991; Wong et al., 1998; Wang et al., 2008; Sasaki et al., 2009a). The NOX homologs, NOX1, and NOX2, complementarily generate ROS during osteoclastogenesis (Sasaki et al., 2009b).

Several reports have suggested that ROS signaling can lead to activation of mitogen-activated protein kinase (MAPK), phosphoinositide-3 kinase (PI3K), and nuclear factor kappa-light-chain-enhancer of activated B cells (NF-kB) (Thannickal and Fanburg, 2000; Droge, 2002). NF-kB, which mediates IκBα phosphorylation and degradation, was the first transcription factor shown to respond to ROS (Schreck et al., 1991). Pretreatment of osteoclasts with antioxidants, to inhibit oxidation, was shown to reduce RANKL-induced AKT, NF-kB, and extracellular signal-regulated kinase (ERK) activation (Ha et al., 2004). Coincidentally, NF-kB also plays a pivotal role in cytokine-induced periodontal tissue damage (Chapple, 1997). Application of an antioxidant inhibited the responses of osteoclast precursors to RANKL; including activation of c-Jun N-terminal kinase, p38 MAPK and ERK, and inhibited osteoclast differentiation (Lee et al., 2005). RANKL-mediated ROS induces long lasting Ca^2+^ oscillations that activate the transcription factor, nuclear factor of activated T-cells, cytoplasmic 1 (NFATC1) (Kim et al., 2010). Taken together, ROS cause hard tissue destruction via osteoclastogenesis, as well as soft tissue destruction.

## Mechanisms that protect against oxidative stress

**Figure 2:**
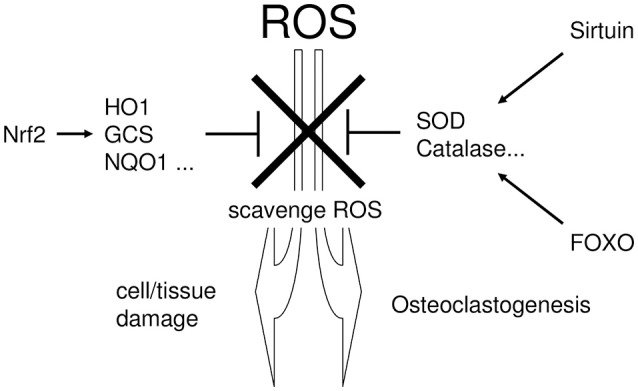
The linking the periodontitis, the regulatory pathways of ROS scavenging enzymes, and the defense mechanism against tissue destruction. Excessive ROS exhibit cytotoxicity and induce tissue destruction in periodontal tissue. To protect such ROS-mediated cytotoxicity, cells possess several regulatory pathways which regulate the production of cytoprotective enzymes that scavenge ROS. NRF2, Sirtuin, and FOXO are situated in the major regulatory pathways for cytoprotective enzyme production.

Cells normally possess several regulatory pathways that protect against oxidative stress (Furukawa-Hibi et al., 2005; Kensler et al., 2007; Hsu et al., 2008), by producing cytoprotective enzymes that scavenge ROS (Mates and Sanchez-Jimenez, 1999). NRF2 (Thimmulappa et al., 2002), Sirtuin (Chen et al., 2011), and FOXO (Liu et al., 2005) are major regulatory pathways for cytoprotective enzymes (Kanzaki et al., 2016a). Figure 2 summarizes the linking the periodontitis, the regulatory pathways of ROS scavenging enzymes, and the defense mechanism against tissue destruction.

NRF2 transcriptionally induces cytoprotective enzymes, such as heme oxygenase-1 (HO-1) (Alam et al., 1999), NAD(P)H: quinone reductase (NQO1) (Favreau and Pickett, 1991), gamma-glutamylcysteine synthetase (GCS) (Wild et al., 1998), and the auxiliary cellular NADPH regenerating enzyme, glucose 6-phosphate dehydrogenase (G6PD) (Thimmulappa et al., 2002). In addition, Sirtuin- and FOXO-induced superoxide dismutase (SOD) (Kops et al., 2002; Nemoto and Finkel, 2002) convert superoxide to hydrogen peroxide (Baehner et al., 1975), which is subsequently detoxified by catalase (CAT) (Essers et al., 2004). These cytoprotective enzymes play a critical role in the scavenging and detoxification of ROS.

Maintaining a balance between ROS and antioxidants is essential for periodontal health. In patients with severe chronic periodontitis, down-regulation of the NRF2 pathway was observed in polymorphonuclear leucocytes (Sima et al., 2016). In addition, neutrophils from patients with severe chronic periodontitis exhibited hyper-reactivity to bacterial stimuli (Dias et al., 2013). Conversely, activation of NRF2 prevented alveolar bone loss in an experimental animal study (Bhattarai et al., 2016). In this context, downregulation of NRF2 pathway might lead to the increase in ROS, which causes tissue destruction.

Cytoprotective enzymes are also thought to interfere with osteoclastogenesis, where ROS functions as an intracellular signaling molecule (Ha et al., 2004). Indeed, RANKL stimulation decreases expression of NRF2-dependent cytoprotective enzymes, thereby facilitating intracellular ROS signaling (Kanzaki et al., 2013, 2016b). Induction of cytoprotective enzymes by NRF2 activation subsequently inhibits osteoclastogenesis (Kanzaki et al., 2013, 2014, 2015; Sakai et al., 2013; Gambari et al., 2014; Lee et al., 2014; Lu et al., 2015; Bhattarai et al., 2016). Inversely, augmented osteoclastogenesis and bone destruction was observed with NRF2 deficiency (Rana et al., 2012; Hyeon et al., 2013; Ibanez et al., 2014; Lippross et al., 2014; Sun et al., 2015). Taken together, this shows that NRF2-dependent cytoprotective enzymes play a critical role in the regulation of bone destruction.

Enlargement of periapical lesions of the teeth in experimental animals has been associated with decreased expression of Sirtuin (SIRT6), causing increased apoptosis of osteoblasts (Kok et al., 2015). Activation of Sirtuin, by resveratrol (Tamaki et al., 2014) or overexpression of SIRT6 in osteoblasts by lentiviral gene transfer (Kok et al., 2015; Hou et al., 2016), inhibited bone destruction. Therefore, not only NRF2- but also Sirtuin-mediated cytoprotective mechanisms control periodontal tissue homeostasis.

Activated FOXOs enhance antioxidant defense by augmenting cytoprotective enzymes, which successfully prevent inflammation and bone destruction (Chung et al., 2011; Kousteni, 2011). FOXO activation directly inhibits osteoclastogenesis (Bartell et al., 2014; Tan et al., 2015). However, FOXO binds to β-catenin, which exhibits inhibitory effects on osteoblastic differentiation. Thus, continuous stimulation of the FOXO/β-catenin pathway may reduce bone formation (Galli et al., 2011). In addition, *porphyromonas gingivalis*-induced ROS activate FOXO transcription factors through JNK signaling, which resulted in the FOXO1-controlled oxidative stress responses, such as inflammatory cytokine production and cell survival (Wang et al., 2015).

A relationship between cytoprotective enzymes themselves and periodontitis has also been reported. SOD is one of the most extensively investigated enzymes, and is closely associated with periodontitis. A positive relationship was demonstrated between the progression of periodontitis and serum SOD concentrations in experimental animals (Sobaniec and Sobaniec-Lotowska, 2000). This has been confirmed in human gingival crevicular fluid (GCF) (Akalin et al., 2005; Wei et al., 2010), serum (Wei et al., 2010), and saliva SOD (Canakci et al., 2009; Guentsch et al., 2012; Karim et al., 2012). Clinical studies in humans indicate that periodontal therapy returns elevated SOD levels to normal (Novakovic et al., 2013, 2014; Sukhtankar et al., 2013; Singh et al., 2014). Induction of SOD inhibited experimental periodontitis in animals (Petelin et al., 2000) and augmentation of anti-oxidant capacity by nutrient supplementation positively affected periodontal therapy in clinical trials (Biju et al., 2014; Daiya et al., 2014; Muniz et al., 2015). Furthermore, genetic mutation of *SOD* is considered to be a risk factor for periodontitis (Kazemi et al., 2015). These data support a role for SOD as a potential diagnostic marker for periodontitis. Therapeutically targeting SOD for the treatment of periodontitis may however produce substantive side effects, due to its beneficial effect on periodontopathogenic bacteria. SOD exhibited protective effects on the periodontopathogenic anaerobes *Porphyromonas gingivalis* (Lynch and Kuramitsu, 1999) and *Aggregatibacter actinomycetemcomitans* (Balashova et al., 2007).

HO-1 is another cytoprotective enzyme involved in the pathology of periodontitis. HO-1 inhibited RANKL upregulation in human cultured periodontal ligament cells (Lee et al., 2010) and lipopolysaccharide-induced production of proinflammatory mediators in cultured macrophages (Choi et al., 2014). Immunohistochemistry demonstrated that HO-1 was broadly expressed in periodontal tissue with chronic periodontitis (Gayathri et al., 2014). G6PD expression was also increased in the gingiva of patients with gingivitis (Di Paola et al., 2005; Yu et al., 2015). These data support the concept that the protective mechanisms provided by cytoprotective enzymes against ROS also play an inhibitory role in the progression of tissue destruction.

Clinically, it has been reported that markers of oxidative stress in saliva could serve as diagnostic markers for periodontitis (Sawamoto et al., 2005; Almerich-Silla et al., 2015; Banasova et al., 2015; Tothova et al., 2015) and therapeutic intervention against experimental periodontitis in rats improved total serum antioxidant levels (Saglam et al., 2015). A better understanding of the mechanisms that protect against oxidative stress will be useful for gaining a precise understanding of periodontitis pathology, which in turn, will contribute to the development of therapies for periodontitis treatment.

## Summary and perspective

In this review, we have summarized recent evidence of the relationship between oxidative stress and periodontitis, a disease in which oxidative stress is both directly and indirectly involved with tissue destruction. Many reports describe a relationship between oxidative stress and periodontitis, with the balance between oxidative stress and defense mechanisms characterizing the pathological condition of the periodontitis. As such, some enzymes that are cytoprotective against oxidative stress could serve as diagnostic markers for periodontitis. Furthermore, a strong potential therapeutic approach for periodontitis would be to augment enzymes that protect against oxidative stress.

Although oxidative stress in periodontitis has been extensively investigated, there remains little information about the relationship between tissue damage and the immunological reactions exerted via ROS production in immune cells. Further investigation in this area is urgently needed for a comprehensive understanding of periodontitis.

## Author contributions

Conception and design: HK and YN. Search references: SW, TN, YY, YN, YK, KI, SF, and YM. Drafted manuscript: HK, SW, YY, and YN. Critically revised the manuscript: HK, SW, YY, YK, KI, SF, YM, and YN.

### Conflict of interest statement

The authors declare that the research was conducted in the absence of any commercial or financial relationships that could be construed as a potential conflict of interest.
